# The global pendulum swing towards community health workers in low- and middle-income countries: a scoping review of trends, geographical distribution and programmatic orientations, 2005 to 2014

**DOI:** 10.1186/s12960-016-0163-2

**Published:** 2016-10-26

**Authors:** Helen Schneider, Dickson Okello, Uta Lehmann

**Affiliations:** 1 School of Public Health & SAMRC/UWC Health Services to Systems Unit, University of the Western Cape, Robert Sobukwe Road, Bellville, Cape Town 7535 South Africa; 2 Health Policy and Systems Division, School of Public Health and Family Medicine, University of Cape Town, Anzio Road, Observatory, Cape Town, 7925 South Africa; 3 School of Public Health, University of the Western Cape, Robert Sobukwe Road, Bellville, Cape Town 7535 South Africa

**Keywords:** Community health worker, Lay health worker, Village health worker, Maternal-child health, Community health worker programmes, Integrated community case management

## Abstract

**Background:**

There has been a substantial increase in publications and interest in community health workers (CHWs) in low- and middle-income countries (LMIC) over the last years. This paper examines the growth, geographical distribution and programmatic orientations of the indexed literature on CHWs in LMIC over a 10-year period.

**Methods:**

A scoping review of publications on CHWs from 2005 to 2014 was conducted. Using an inclusive list of terms, we searched seven databases (including MEDLINE, CINAHL, Cochrane) for all English-language publications on CHWs in LMIC. Two authors independently screened titles/abstracts, downloading full-text publications meeting inclusion criteria. These were coded in an Excel spreadsheet by year, type of publication (e.g. review, empirical), country, region, programmatic orientation (e.g. maternal-child health, HIV/AIDS, comprehensive) and CHW roles (e.g. prevention, treatment) and further analysed in Stata14. Drawing principally on the subset of review articles, specific roles within programme areas were identified and grouped.

**Findings:**

Six hundred seventy-eight publications from 46 countries on CHWs were inventoried over the 10-year period. There was a sevenfold increase in annual number of publications from 23 in 2005 to 156 in 2014. Half the publications were reporting on initiatives in Africa, a third from Asia and 11 % from the Americas (mostly Brazil). The largest single focus and driver of the growth in publications was on CHW roles in meeting the Millennium Development Goals of maternal, child and neonatal survival (35 % of total), followed by HIV/AIDS (16 %), reproductive health (6 %), non-communicable diseases (4 %) and mental health (4 %). Only 17 % of the publications approached CHW roles in an integrated fashion. There were also distinct regional (and sometimes country) profiles, reflecting different histories and programme traditions.

**Conclusions:**

The growth in literature on CHWs provides empirical evidence of ever-increasing expectations for addressing health burdens through community-based action. This literature has a strong disease- or programme-specific orientation, raising important questions for the design and sustainable delivery of integrated national programmes.

**Electronic supplementary material:**

The online version of this article (doi:10.1186/s12960-016-0163-2) contains supplementary material, which is available to authorized users.

## Background

As has now been noted by many, the Millennium Development Goal (MDG) era saw a global resurgence of interest in the role of community health workers (CHWs) in health systems, an interest that is set to continue in new global health agendas [1]. The mobilization of international funding from bilateral, multilateral and private philanthropic sources has greatly increased investment in programmes to meet the MDG targets. Further, through the popularization of the concept of “task shifting”, the involvement of lay and community health workers has emerged as a rational strategy for addressing the vast shortfall in human resources impeding the roll-out of programmes in many countries.

A feature of countries that made the most progress in the health of their populations has been their investment in strategies that engage households and communities directly as part of primary health care [2]. An expanding list of countries with large-scale and stable CHW programmes and a growing evidence base on the effectiveness of CHWs in achieving specific health outcomes [3–6] have brought renewed global confidence in CHWs. A number of significant international consensus statements have recommended that CHW programmes be integrated into health systems, increasingly linking these to the concept of universal health coverage (UHC) [7–9].

Driven by different imperatives and needs, CHW initiatives have taken a variety of regional- and country-specific forms. Some, such as the Brazilian *Programa Saúde da Famiília*, Ethiopia’s health extension workers and the *Behvarzs* of Iran, have been part of broader social, political and health sector change. In several Asian countries (Pakistan, Bangladesh, Nepal), CHW programmes have been established in response to the public health challenge of high maternal, neonatal and under-5 mortality. In the HIV-affected countries of southern Africa, home-based care and support emerged organically through local community and non-governmental organizations as a response to overwhelming care and social needs. In other African countries, Global Health Initiatives and partnerships focused on malaria and childhood illness have been influential. CHWs and CHW programmes are thus a broad umbrella concept and practice under which a diverse array of programmatic priorities, roles and forms of community involvement in health and health care delivery exist.

How are this diversity and the global pendulum swing towards CHWs reflected in the research on CHWs and CHW programmes? We report on a scoping review of trends, geographical distribution and programmatic orientations in the indexed literature on CHWs in low- and middle-income countries over a 10-year period (2005–2014). A scoping review aims to “map the existing literature in a field of interest in terms of the volume, nature and characteristics of the primary research” [10]. The purpose of this review is thus not to appraise or synthesize the evidence base for effectiveness, feasibility or impact of CHWs, or to assess the quality of research, but rather to present a descriptive account of the contours of a rapidly evolving and heterogenous field.

Specifically, we were animated by the following questions:

1. What are the trends in numbers of publications on CHWs?
2. Which countries and regions are represented in these trends?
3. What is the profile of health programmes and global health agendas (e.g. maternal-child health, HIV/TB)?
4. What types of CHW roles (e.g. prevention, treatment, social mobilization) are being foregrounded?
5. What does it suggest for future thinking on CHW programmes?

Our definition of a CHW for the purpose of this review is that proposed by Naimoli et al. [11] as “a health worker who receives standardized training outside the formal nursing or medical curricula to deliver a range of basic health, promotional, educational, and outreach services, and who has a defined role within the community system and larger health system.” In this review, we focus on those cadres whose activities are primarily community- rather than facility based.

## Methods

### Scoping review methodology

The review methodology followed broadly the steps proposed by Levac et al. [12]. HS and UL developed the scoping review questions (and coding schemes) based on previous literature reviews. After an initial search, the volume of new literature in recent years immediately became apparent. Since we were primarily interested in trends and patterns, we decided to limit the scope of the search to the indexed literature and focus on a 10-year period. In October 2015, we searched the following electronic databases through EBSCOhost: Academic Search Premier, Africa-Wide Information, CINAHL, PsycINFO, SocINDEX and MEDLINE, for all English-language publications on CHWs, indexed from 2005 to 2014, and in countries defined by the World Bank as low- and middle income (http://data.worldbank.org/news/new-country-classifications-2015). We also searched the Cochrane database for systematic reviews on CHWs.

Recognizing the wide diversity of forms and titles of CHWs across the globe, we developed an inclusive list of search terms (Table 1). However, we deliberately excluded certain terms, such as traditional birth attendants and facility-based lay counsellors, as these would have touched on significant other bodies of literature.

**Table 1:** Search terms and inclusion/exclusion criteria

Search terms	“community health worker*” OR “volunteer health worker*” OR “lay health worker*” OR “lay health advis*r” OR “lay health advis*rs” OR “lay health educator*” OR “village health worker*” OR “village health volunteer*” OR “lady health worker*” OR “community health volunteer*” OR “community health agent*” OR “community health promotion” OR “community health promoter*” OR “community health aide*” OR “health assistant worker*” OR “home based care” OR “home community based care*” OR “community health agent*” OR “health surveillance assistant*” OR “community care giver*” OR “community caregiver” OR “accredited social health activists” OR “asha” OR “mitanins” OR “mitanin” OR “family health team*” OR “family health program*” OR “integrated community case management” OR “ICCM”
Inclusion/exclusion criteria	English-language publications
	Low- and middle-income countries
	Empirical findings, reviews, trial protocols, extended analyses, scientific letters and conference proceedings
	Not the following:
	• Editorials, letters, short commentaries, news items
	• Traditional birth attendants and traditional healers
	• Facility-based cadres, such as lay counsellors
	• Family care givers, peer supporters or counsellors, expert patients
	• Community medicine retailers/sellers
	• Community rehabilitation workers
	• CHWs as field workers for research
	• CHWs as a recommendations but not a focus of the findings
	• Household surveys describing utilization of different providers, including CHWs

The search was conducted sequentially with all the EBSCOhost databases except MEDLINE searched together in the first step, followed by the search of MEDLINE in a second step. Each step yielded 5635 and 1445 hits, respectively.

From October 2015 to January 2016, two authors (HS and DO) independently screened the titles and abstracts obtained in these searches, based on the inclusion criteria (Table 1). In this initial process, we selected a total of 897 publications, which were entered in a database (Mendeley) and full texts downloaded. Entries were then independently coded by two authors (HS and DO) in an Excel spreadsheet following the scheme outlined in Table 2. In an iterative process that involved removing publications that did not meet the inclusion criteria, and adding relevant publications identified in the subset of review papers, a final total of 678 publications was selected for analysis.

**Table 2:** Coding scheme for extracted papers

Theme	Code
Programmatic focus	Maternal-child health (MCH)
	HIV/TB
	Malaria
	Reproductive health
	Non-communicable diseases
	Mental health
	Other
	Comprehensive (two or more of the above)
Role	Treatment
	Prevention and promotion (including advocacy and social mobilization)
	Care, counselling, adherence
	Screening, referral, mediating access
	Two or more of the above

Coding relied on the abstract in the first instance, with further verification based on the full-length article, if the abstract was not sufficient. We categorized each entry into year of publication, country and region, and type of publication—empirical, review or “analysis”. Empirical pieces reported research findings (qualitative or quantitative), and reviews were formal appraisals of the literature based on an identifiable search strategy. Some papers used “review” in the title in a more colloquial sense but were substantive reflections or commentaries, drawing on the literature, but not adopting a structured review strategy. We categorized these papers as “analyses”. Publications were also categorized by programmatic focus based on the conventionally accepted approaches (such as maternal-child health, malaria, reproductive health, comprehensive), drawing firstly on the title and abstract, and if this was not stated by scanning the full-length paper for a description of CHW roles. Additional file 1: Table S1 gives a detailed breakdown of the items included under each of the codes. We also noted the type of role such as treatment or prevention or both—performed by the CHWs. The coded items in the Excel spreadsheet were imported into Stata (Version 14) for descriptive quantitative analysis. A qualitative, thematic analysis of key roles within each programmatic area was done, drawing on the subset of review and multi-country articles in the first instance, followed by reading of individual papers if the reviews were judged not sufficient.

### Limitations

The findings reported are not a full inventory of all research and publications on CHWs but rather the trends and patterns of a delimited body of literature in the field, through one search process. Given the volume of publications, we did not conduct a grey literature search. However, we recognize there are significant and influential publications [9, 13–15] and consensus statements [7, 16] in this sphere, whose insights have not necessarily made their way into the indexed literature.

Choices were made in the classification of the paper’s programmatic focus. For example, the prevention of mother to child transmission of HIV (PMTCT) interventions, because they overlap with general maternal health (breastfeeding, antenatal care), were classified under maternal-child health (MCH) rather than HIV/TB. On the other hand, studies evaluating intermittent preventive treatment of malaria in children (IPTc) were classified under malaria because they most often emerged from a malaria programmatic focus. However, integrated community case management interventions, combining pneumonia and malaria treatment of children, were classified under MCH. The specific choices are reflected in Additional file 1: Table S1.

To limit the scope of the review, we also excluded facility-based cadres as it would have meant assessing a growing body of work on task shifting within health facilities, especially in HIV-affected countries where lay counsellors have become an integral part of primary health care teams. Unfortunately, this also excluded significant developments in the field of mental health (see, for example, [17, 18]).

This review does not address a key preoccupation in the literature on the support and systems dimensions of CHW programmes, such as supervision, retention, motivation, monitoring and financing of CHWs.

## Findings

### Overall profile, trends and geographical distribution

**Fig. 1:**
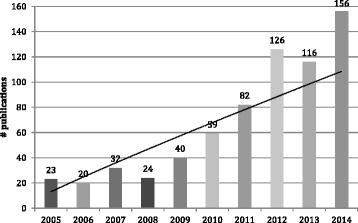
Numbers of indexed publications on CHWs 2005–2014 (*n* = 678)

Of the 678 papers, 604 (89 %) were empirical pieces, 55 (8 %) were reviews and 19 (3 %) were analyses. There was a nearly sevenfold growth in annual number of publications over the period, from 23 in 2005 to 156 in 2014 (Fig. 1).

The papers reported experiences in 46 countries (Additional file 2: Figure S1), with 17 countries contributing at least 10 publications each, amongst them the globally recognized national CHW initiatives (Table 3). Half of the publications came from the Africa Region, just under a third from the Asia/Pacific Region, and 6.5 % had a global perspective. Iran was the only country contributing experiences from the Middle East Region, and the Brazilian programme accounted for 80 % of publications from Latin America, possibly reflecting the English-language bias of the review (Additional file 3: Table S2). Three middle-income countries—South Africa, India and Brazil—each contributed 60 or more papers, together making up 30 % of the total publications.

**Table 3:** Profiles of publications on CHWs in LMIC, 2005–2014

Characteristic		Number	Percent
Type	Empirical	604	89.1
	Review	55	8.1
	Analysis	19	2.8
Region	Africa	345	50.9
	Asia/Pacific	202	29.8
	Americas	75	11.1
	Middle East	12	1.8
	Cross-regional	44	6.5
Countries with 10 or more publications (with name of main CHW cadre)	India (accredited social health activist)	70	10.3
	South Africa	71	10.5
	Brazil (community health agent)	60	8.8
	Ethiopia (health extension worker)	39	
	Uganda (village health teams)	34	
	Malawi (health surveillance assistant)	32	
	Pakistan (lady health worker)	32	
	Kenya	31	
	Bangladesh (*Shasthya Shebika* (BRAC))	28	
	Zambia (community health assistant)	20	
	Nepal (female community health volunteer)	19	
	Ghana	17	
	Tanzania	16	
	Nigeria	14	
	Thailand (community health volunteer)	12	
	Iran (*behvarz*)	12	
	Rwanda (binome)	11	
	Total	518	76.4
Programmatic orientation of publications	MCH	235	34.7
	Comprehensive	116	17.1
	HIV/TB	106	15.6
	Malaria	69	10.2
	Reproductive health	37	5.5
	Non-communicable diseases	30	4.4
	Mental health	28	4.1
	Other	39	5.8
	Not specified	18	2.7
Programmatic orientation of reviews (*n* = 55)	MCH	21	38.2
	Comprehensive	11	20.0
	HIV/TB	6	10.9
	Malaria	4	7.3
	Mental health	4	7.3
	Reproductive health	1	1.8
	Other	3	5.5
	Not specified (system-strengthening focus)	5	9.1

### Programmatic focus

The profile of programmatic foci in the publications, by region and country, is summarized in Table 3 and provided in full in Additional file 3: Table S2.

#### Maternal-child health focus

**Fig. 2:**
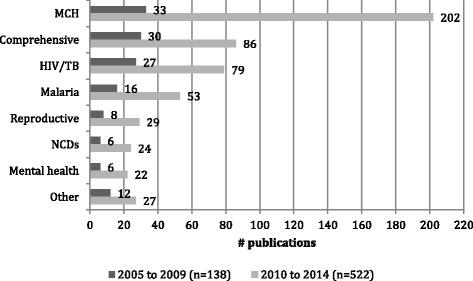
Publication numbers by programme area 2005–2009 and 2010–2014 (excluding “not specified”) (*n* = 660)

By far the most commonly reported CHW roles were those focused on maternal-child health (MCH), accounting for over a third of the total papers as well as the subset of reviews. When comparing the first and second halves of the review period, MCH was also the biggest driver of growth in publications (Fig. 2). The global emergence and promotion of integrated community case management (iCCM) of childhood illness, particularly in Africa, is the single most important element in this. iCCM is a community and CHW-based child survival strategy, adopted by WHO and UNICEF [16]. Three special editions on iCCM were produced in the review period, one in 2012 (*American Journal of Tropical Medicine and Hygiene*) and two in 2014 (*Ethiopian Medical Journal*, *Journal of Global Health*), accounting for the spikes in publications in those 2 years (Fig. 1).

CHW roles in MCH were clustered into three broad areas:

- Maternal and newborn health, including birth preparedness and distribution of misoprostol to prevent post partum haemorrhage in home deliveries [19, 20]; postnatal home visiting, umbilical cord care, thermal care, promotion of exclusive breast feeding and treatment of neonatal infection; [21–23] and support to mothers and infants for the prevention of mother to child transmission of HIV [24–26].
- Promotion of child health, including uptake of immunization [27]; nutrition, including breast feeding, micronutrient supplementation and supplemental feeding [28]; community management of malnutrition [29]; and early childhood development [30, 31].
- Treatment of childhood illness [32, 33] in particular the iCCM strategy [34]. iCCM combines the diagnosis and treatment of malaria with artemisinin combination therapy (ACT), pneumonia with oral antibiotics and diarrhoea with zinc and oral rehydration salts (ORS). It has been facilitated by the development of rapid diagnostic tests (RDTs) for malaria, thus allowing for more accurate diagnosis of fever in young children.

iCCM has been promoted by WHO and UNICEF across sub-Saharan Africa and integrated to varying degrees in country CHW initiatives [35]. It was the most common theme in comparative or cross-country publications from Africa (19 out of 34 papers). While MCH was also a dominant focus of CHW studies from Asia, these programmes were orientated to maternal and newborn health and were more preventive and promotive in approach. They also tended to be tailored programmes, developed in context-specific ways and involving a greater level of community mobilization and participation in their design [36]. In contrast to the African continent, there were no multi-country empirical studies from Asia.

#### Comprehensive focus

Seventeen percent of publications approached CHW roles comprehensively. Publications in this category included systematic reviews addressing the effectiveness of CHWs across a number of programmatic areas, including maternal-child health, HIV and TB [3, 6]. They also included reports or evaluations of provincial or national programmes, amongst them the recognized ones listed in Table 3 (see, for example, [37–40]).

In general, the comprehensive programmes were large-scale government initiatives that combined disease/programme-specific tasks with social, environmental and health surveillance roles. The activities spanned prevention, promotion, treatment and community mobilization. Table 4 outlines the roles of three typical cadres, the health extension workers (HEWs) in Ethiopia, the health surveillance assistant (HSAs) in Malawi and the *Behvarzs* in Iran. These cadres are government employed and receive basic training ranging from 3 months (HSA) to 1 year (HEW) and 2 years (*Behvarz*).

**Table 4:** CHW roles in comprehensive national programmes

Health extension worker (source: [97])	Health surveillance assistance (sources: compiled from [41, 98])	*Behvarz* (source: [99])
Family health: • Family planning • Maternal, newborn and child health • Nutrition • Vaccination Disease prevention and control: • HIV/AIDS and STDs • Tuberculosis • Malaria • First aid Hygiene and environmental sanitation: • Construction and maintenance of sanitary latrines • Solid and liquid waste disposal • Water supply safety • Control of insects and rodents • Food hygiene and safety • Personal hygiene • Healthy home environment Health education and communication	Environmental health: • Hygiene, sanitation and water supply • Disease surveillance and outbreak response • Vector and vermin control Maternal-child health: • Immunization, vitamin A, de-worming; growth monitoring, nutritional supplementation, tetanus vaccination; • Integrated community case management Reproductive health: • Condoms and oral and injectable contraceptives HIV/TB: • Testing/screening, follow-up, drug dispensing Treatment and referral of minor ailments Community education Training and supervision of village health councils	Annual census Providing basic health care: • Maternal and child health, delivery • Family planning • Oral health • Youth and elderly health • Community-based rehabilitation Preventive activities: • Health education, screening Disease management: • Communicable and non-communicable diseases School health: • School environment • Screening Environmental health: • Food safety, sanitation, safe water Occupational health Collaborations: • Rural health councils • Family doctors Promoting community participation and inter-sectoral collaboration

As the evidence base on CHW roles and access to diagnostic and treatment technologies expand, a key risk in comprehensive programmes is role overload. A number of papers addressed this, outlining the need to maintain realistic expectations and workloads of CHWs and proposing new ways of configuring community-based services, such as specialization of functions and a division of labour [41, 42]. Related to this is the ongoing preoccupation with maintaining an appropriate balance between prevention, treatment, facilitating access and community mobilization [43].

#### Other programmatic foci

The next biggest programmatic focus of publications was on HIV/AIDS and TB (16 %). More than three quarters (76 %) of the publications with this focus came from the heavily AIDS-affected countries of sub-Saharan Africa (particularly South Africa) (Additional file 3: Table S2). The CHW roles in HIV/AIDS and TB were mostly oriented towards care, counselling, adherence and social support and promoting patient self-management, with some elements of prevention and promotion [44]. In an earlier period, they were focused on palliative home-based care and on implementing the WHO-advocated “DOTS” (Directly Observed, Short Course treatment) for TB [45, 46]. With the advent of antiretroviral therapy, roles shifted towards home-based HIV testing; referral for, or home initiation of, antiretroviral therapy (ART); and community-based adherence support and follow-up of care for ART [47, 48] and TB treatment [49], increasingly as integrated programmes [50, 51]. In a number of countries, the mobilization of community health workers for HIV/AIDS appears to have emerged as a parallel development alongside other programmatic initiatives, producing a mixed profile of lay health work and posing challenges of local coordination and integration [52, 53].

Ten percent of publications reported on the role of CHWs in the control of malaria (apart from their contribution to iCCM). These included community case management or home management of malaria with or without the use of rapid diagnostic tests [54], distribution of intermittent preventive treatment (IPT) to pregnant women and children [55–57] and the promotion of insecticide-impregnated bed nets [58].

CHWs have long established roles in family planning (often referred to as community-based distributors) and commonly provided as vertical programmes [59]. Several papers reported on experiences with CHWs providing injectable contraceptives [4], including the more recent contraceptive implants (Implanon) [60]. CHWs have also been involved in promoting cervical [61] and breast cancer screening [62, 63].

Other established specialist CHW roles reported in the period include the distributors of ivermectin to treat river blindness in the “community-directed interventions” [64] developed by the WHO/TDR-supported African Programme for Onchocerciasis Control. Similar roles were also reported for other “neglected tropical diseases” such as schistosomiasis [65] and trachoma [66]. CHWs were also deployed in the early detection of Buruli ulcer [67] and visceral leishmaniasis [68] in high-burden areas.

#### Emerging programme foci

Reflecting changing demographic and epidemiological profiles, a small but steady number of publications across years and regions addressed CHW roles in non-communicable diseases. They included primary preventive programmes for cardiovascular disease and diabetes, focusing on lifestyle risk factors such as physical activity, diet and smoking cessation in Thailand [69], India [70], Pakistan [71], Brazil [72] and Ghana [73]; community-based screening, referral and follow-up in Kenya [74], South Africa [75], Iran [76], Brazil [77] and Pakistan [78]; and population surveillance for NCDs in India [79]. There were no reviews within or across low- and middle-income countries (LMIC) of CHW roles in chronic disease care in the period.

A number of empirical papers and reviews reported on the integration of mental health into existing CHW initiatives [5]. In Pakistan, Lady Health Workers successfully provided cognitive-based therapies for perinatal depression [80]. In Malawi and Kenya, CHWs were given general training in mental health awareness, identification and family support [81, 82]. In Brazil, community health agents screened for dementia and depression in the elderly [83, 84]. In India, community-based care for schizophrenia and dementia sufferers was evaluated as part of “collaborative care” (in a team with professionals) [85, 86].

### Regional variations

**Fig. 3:**
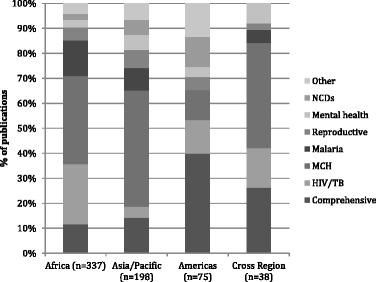
Regional profile of programmatic orientations (excluding the Middle East and “not specified”, *n* = 648)

Programmatic emphases varied between regions (Fig. 3). In Africa, with high burdens of malaria and HIV, publications were more evenly distributed between HIV/TB, malaria and MCH. In Asia, MCH dominated as a programmatic focus, although with significant nodes of development in mental health and NCDs. The comprehensive orientation of the Latin American publications reflects the influence of the Brazilian Family Health Programme, which is delivered with the close support of health professionals and primary health care facilities.

### Types of roles

**Fig. 4:**
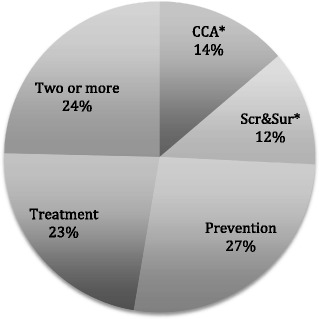
Role orientations of CHWs in papers (*n* = 615). *CCA* care, counselling, adherence; *Scr&Sur* screening, surveillance, referral

Where this was identifiable (*n* = 615 papers), the roles of CHWs along the promotion-prevention-treatment continuum were coded. These were broadly clustered in four areas (Fig. 4): (1) diagnosis and treatment (notably in iCCM and malaria); (2) prevention and promotion, spanning the distribution of preventive technologies (such as contraceptives), to education (such as newborn health, breast feeding), to processes of social mobilization; (3) screening, referral and surveillance activities, such as early detection of cancers or chronic disease; and (4) counselling, care and adherence support for adults receiving treatment for chronic conditions (such as HIV/TB, mental illness). One quarter of the papers reported roles spanning two or more of these areas, with several papers suggesting the importance of combined roles in the community legitimacy of CHWs [43, 87].

### Within-country plurality

Publications most often focused on evaluating or describing the work of one type of CHW. However, even in countries with recognized national programmes, the papers from this country, when brought together as a collection, portrayed a more diverse reality. In Ethiopia, for example, where the health extension workers (HEWs) are the recognized CHWs, at least two other community-based workers in communities were described: the community-based reproductive agents delivering contraceptive technologies [88] and community AIDS volunteers linked to NGOs and ART treatment programmes [53, 89]. Within the Health Extension Programme itself, HEWs relate to a cascade of community actors: they mobilize volunteer community health workers, also referred to as the Health Development Army, who, in turn, nominate female household members for training as “model households” [39, 90].

In Uganda, where community health workers are volunteers, and where roles and functions were less clearly defined nationally, the 34 papers in the collection described a plethora of disease- or programme-specific workers and interventions, including iCCM, maternal and new born health, reproductive health, malaria, onchocerciasis, antiretroviral therapy for HIV and palliative care.

Standing and Chowdhury [91] describe how community health workers in Bangladesh are positioned in dense and plural local health care environments, where they are but one player amongst the many informal, formal and traditional sources of care and healing which community members draw on. In such contexts, CHWs play a variety of different roles—as a generic provider linked to an agency (such as Building Resources Across Communities (BRAC)), specialized workers (e.g. reproductive health distributors), as agents that mediate relationships between households and the formal health system or as expert patients.

## Discussion

There has been a large growth in publications on CHWs in recent years, most notably since 2011. This growth has been driven by the MDGs, especially those related to child survival, which have placed heavy emphasis on community-based activities. The integrated community case management strategy, in particular, was the product of a concerted global agenda setting process by an “epistemic community” of international NGOs, multilateral and bilateral agencies and academic actors, who developed and promoted a package of feasible interventions targeted at the major causes of child mortality [92].

Despite the extensive reliance on lay heath workers and greater levels of international funding flowing to HIV/AIDS [93], there were fewer publications, whether empirical, comparative or review, addressing this programme area. There are a number of possible reasons for this: the review period may have missed an earlier generation of publications on community caregivers and counsellors; strategies such as the “community system strengthening” framework of the Global Fund for AIDS, TB and Malaria [94] and UNAIDS’ “90-90-90” treatment targets [95] have not focused specifically on CHWs as players; HIV-treatment programmes tend to be facility based; and the HIV response also has a shorter history than the child survival interventions, which evolved into the iCCM package in an iterative process over many years and which built on a long-standing MCH focus in primary health care.

As low- and middle-income countries confront a new generation of health challenges such as non-communicable diseases, mental health and violence and injury, the repertoire of possible CHW roles is ever-expanding. There is a danger of role fragmentation and overload and a need to re-think roles in new and more complex ways. Layered approaches where roles are distributed amongst a number of cadres from expert patient to volunteer to remunerated cadres may be required [39, 91]. Similarly, strategies of specialization [41] and the balance between disease-specific and integrated approaches need to be defined. In the process, there is a risk that the social and environmental health roles of CHWs get crowded out by technical and treatment roles of core cadres, especially if the latter are incentivized [43].

The CHW programmes and interventions reported also reflected different orientations along a continuum of technical/biomedical to social/participatory and with different mixes of prevention, promotion, treatment and social mobilization. Some approached CHW roles as a set of predefined intervention packages, while in others CHW roles emerged as tailored programmes specific to local and national contexts, sometimes developed through action-learning methodologies. These differences suggest different kinds of relationship to community. They tended to follow regional and country lines indicating their different histories, programmatic traditions and discourses. This is worthy of further examination.

Similarly, the initiatives reported had varying degrees of closeness to government and the formal health system. Most LMIC health systems have experimented with and developed policy on CHWs. However, the extent to which reports (whether programme specific or comprehensive) were embedded in or reported on official, national CHW programmes varied considerably. As the number of initiatives grows, the need for national and local coordination and stewardship becomes more urgent. While some of the papers touched on these broader system questions, it is beyond the scope of this paper to discuss these.

## Conclusions

The growth in literature on CHWs provides empirical evidence of increasing expectations for addressing health burdens through CHWs and community-based action. However, as Tulenko et al. point out, these developments have been heavily donor dependent, resulting in a fragmented environment where disease-specific responses dominate [8]. This raises important questions of sustainability and the need to integrate the plethora of new initiatives into coherent national programmes and local primary health care systems [35, 96].

## Additional files

Additional file 1: Table S1. Coding of papers by theme. (DOCX 16 kb)
Additional file 2: Figure S1. Distribution of publications by region and country. (DOCX 15 kb)
Additional file 3: Table S2. Distribution of publications by region, country and programmatic focus. (DOCX 77 kb)

## Abbreviations

ART: Antiretroviral therapy
BRAC: Building Resources Across Communities (formerly Bangladesh Rural Advancement Committee)
CHW: Community health worker
HEW: Health extension worker
iCCM: Integrated community case management
LMIC: Low- and middle-income countries
MCH: Maternal-child health
NCD: Non-communicable disease
