# Supplementary material for: The global pendulum swing towards community health workers in low- and middle-income countries: a scoping review of trends, geographical distribution and programmatic orientations, 2005 to 2014
Source: Hum Resour Health. 2016 Oct 26;14:65. doi: 10.1186/s12960-016-0163-2 (PMC5081930; doi:10.1186/s12960-016-0163-2)
Supplement: Additional file 1: Table S1. — Coding of papers by theme. (DOCX 16 kb) [file 12960_2016_163_MOESM1_ESM.docx]

Additional Table 1: Coding of papers by theme

| Theme | Code | Activities included under the code |
| --- | --- | --- |
| Program-matic focus | MCH | Integrated community case management of childhood illness (pneumonia, diarrhoea and malaria)  Community case management of pneumonia  Antenatal care and education, birth preparedness  Distribution of misoprostol to prevent post partum haemorrhage and for post abortion care  Post natal/neonatal interventions: prevention, screening and referral or treatment, umbilical cord care, treatment of omphalitis, prevention of hypothermia  Nutrition: promotion of exclusive breastfeeding, community management of malnutrition (ready to use therapeutic foods), micro-nutrient supplements, education on complementary feeding  Prevention of mother-to-child transmission of HIV  Childhood immunisation, Hepatitis B vaccination  Early childhood development  Responsive stimulation, attachment  Screening for foetal alcohol syndrome |
|  | HIV/TB | Home based counseling  Home based HIV testing  Home based ART initiation and follow up  Screening and referral for TB and HIV treatment  Adherence support  Condom distribution |
|  | Malaria | Home management of malaria with artemisinin combination therapy  Intermittent preventive treatment  Distribution of insecticide-treated bed nets |
|  | Reproductive health | Community based distributors of Depo medroxyprogesterone acetate (DPMA) and  Implanon  Cervical and breast cancer screening |
|  | Non communic-able diseases | Prevention of risk factors from lifestyle, including exercise, diet and smoking cessation  Early detection through screening, referral and community based follow-up  Secondary prevention of complications of disease such as stroke and myocardial infarction  Household surveillance of NCDs |
|  | Mental health | Depression in peri-natal period and in elderly  Household follow up of schizophrenia  Screening and referral for dementia  Screening and referral for epilepsy |
|  | Other | Mass treatment of onchocerciasis, trachoma, schistosomiasis  Distribution of insecticide treated bednets for visceral leishmaniasis  Strategies to address Tungiasis, Kala-azar, Dengue fever, Buruli ulcer, guinea worm  Sanitation, water, waste disposal, cholera prevention  Oral health promotion in children  Eye health; corneal injury and fungal infections  Ear health and hearing  Injury management and prevention; promotion of home safety, Family violence, Disaster preparedness  Adolescent health, Health promoting schools  Care of elderly |
|  | Comprehen-sive | Packages of integrated interventions involving two or more programmatic areas and a multi-purpose worker |
