# Supplementary material for: The global pendulum swing towards community health workers in low- and middle-income countries: a scoping review of trends, geographical distribution and programmatic orientations, 2005 to 2014
Source: Hum Resour Health. 2016 Oct 26;14:65. doi: 10.1186/s12960-016-0163-2 (PMC5081930; doi:10.1186/s12960-016-0163-2)
Supplement: Additional file 2: Figure S1. — Distribution of publications by region and country. (DOCX 15 kb) [file 12960_2016_163_MOESM2_ESM.docx]

Additional file 2: Figure S1: Distribution of publications by region and country
