# Supplementary material for: The global pendulum swing towards community health workers in low- and middle-income countries: a scoping review of trends, geographical distribution and programmatic orientations, 2005 to 2014
Source: Hum Resour Health. 2016 Oct 26;14:65. doi: 10.1186/s12960-016-0163-2 (PMC5081930; doi:10.1186/s12960-016-0163-2)
Supplement: Additional file 3: Table S2. — Distribution of publications by region, country and programmatic focus. (DOCX 77 kb) [file 12960_2016_163_MOESM3_ESM.docx]

Additional Table 2: Distribution of publications by region, country and programmatic focus

| Region | Country | Comp | HIV/TB | MCH | Malaria | RH | Mental | NCDs | Other | Not spec | Total | % |
| --- | --- | --- | --- | --- | --- | --- | --- | --- | --- | --- | --- | --- |
| Africa | South Africa | 6 | 37 | 9 | 0 | 4 | 4 | 6 | 2 | 3 | 71 |  |
|  | Ethiopia | 7 | 5 | 21 | 2 | 3 | 0 | 0 | 1 | 0 | 39 |  |
|  | Uganda | 1 | 6 | 18 | 6 | 3 | 0 | 0 | 0 | 0 | 34 |  |
|  | Malawi | 5 | 10 | 12 | 2 | 1 | 2 | 0 | 0 | 0 | 32 |  |
|  | Kenya | 7 | 4 | 10 | 3 | 1 | 2 | 1 | 1 | 2 | 31 |  |
|  | Zambia | 3 | 4 | 4 | 8 | 1 | 0 | 0 | 0 | 0 | 20 |  |
|  | Ghana | 3 | 1 | 6 | 4 | 0 | 0 | 1 | 1 | 1 | 17 |  |
|  | Tanzania | 0 | 1 | 8 | 4 | 0 | 0 | 0 | 2 | 1 | 16 |  |
|  | Nigeria | 1 | 1 | 4 | 4 | 1 | 2 | 0 | 1 | 0 | 14 |  |
|  | Rwanda | 3 | 3 | 3 | 1 | 0 | 0 | 0 | 1 | 0 | 11 |  |
|  | Senegal | 1 | 0 | 0 | 6 | 0 | 0 | 0 | 0 | 0 | 7 |  |
|  | Madagascar | 0 | 0 | 0 | 1 | 2 | 0 | 0 | 0 | 0 | 3 |  |
|  | Lesotho | 0 | 2 | 0 | 0 | 0 | 0 | 0 | 0 | 0 | 2 |  |
|  | Cameroon | 0 | 0 | 0 | 0 | 0 | 0 | 0 | 2 | 0 | 2 |  |
|  | DRC | 0 | 0 | 1 | 1 | 0 | 0 | 0 | 0 | 0 | 2 |  |
|  | Sudan | 0 | 0 | 2 | 0 | 0 | 0 | 0 | 0 | 0 | 2 |  |
|  | Liberia | 0 | 0 | 0 | 0 | 1 | 0 | 0 | 0 | 0 | 1 |  |
|  | Angola | 1 | 0 | 0 | 0 | 0 | 0 | 0 | 0 | 0 | 1 |  |
|  | Botswana | 0 | 1 | 0 | 0 | 0 | 0 | 0 | 0 | 0 | 1 |  |
|  | Burkina Faso | 0 | 0 | 0 | 1 | 0 | 0 | 0 | 0 | 0 | 1 |  |
|  | Gambia | 0 | 0 | 0 | 1 | 0 | 0 | 0 | 0 | 0 | 1 |  |
|  | Guinea-Bissau | 0 | 0 | 0 | 0 | 0 | 0 | 0 | 1 | 0 | 1 |  |
|  | Mozambique | 0 | 1 | 0 | 0 | 0 | 0 | 0 | 0 | 0 | 1 |  |
|  | Sierra Leone | 0 | 0 | 1 | 0 | 0 | 0 | 0 | 0 | 0 | 1 |  |
|  | 2 or more countries | 1 | 5 | 20 | 4 | 1 | 0 | 0 | 2 | 1 | 34 |  |
|  | Total | 39 | 81 | 119 | 48 | 18 | 10 | 8 | 14 | 8 | 345 | 50.9 |
|  |  |  |  |  |  |  |  |  |  |  |  |  |
|  |  |  |  |  |  |  |  |  |  |  |  |  |
| Asia/Pacific | India | 14 | 5 | 26 | 4 | 2 | 10 | 2 | 5 | 2 | 70 |  |
|  | Pakistan | 2 | 1 | 19 | 0 | 6 | 1 | 3 | 0 | 0 | 32 |  |
|  | Bangladesh | 2 | 1 | 22 | 0 | 2 | 0 | 0 | 1 | 0 | 28 |  |
|  | Nepal | 0 | 0 | 18 | 0 | 1 | 0 | 0 | 0 | 0 | 19 |  |
|  | Thailand | 3 | 1 | 0 | 0 | 0 | 0 | 6 | 2 | 0 | 12 |  |
|  | China | 3 | 0 | 1 | 0 | 0 | 1 | 1 | 1 | 2 | 9 |  |
|  | Cambodia | 2 | 0 | 0 | 5 | 0 | 0 | 0 | 0 | 0 | 7 |  |
|  | Afghanistan | 1 | 1 | 2 | 0 | 2 | 0 | 0 | 0 | 0 | 6 |  |
|  | Burma | 0 | 0 | 1 | 4 | 0 | 0 | 0 | 1 | 0 | 6 |  |
|  | Vietnam | 0 | 0 | 0 | 2 | 0 | 0 | 0 | 1 | 0 | 3 |  |
|  | Laos | 1 | 0 | 0 | 2 | 0 | 0 | 0 | 0 | 0 | 3 |  |
|  | Indonesia | 0 | 0 | 0 | 1 | 1 | 0 | 0 | 0 | 0 | 2 |  |
|  | Papua New Guinea | 0 | 0 | 1 | 0 | 0 | 0 | 0 | 0 | 0 | 1 |  |
|  | Mongolia | 0 | 0 | 0 | 0 | 0 | 0 | 0 | 1 | 0 | 1 |  |
|  | 2 or more countries | 0 | 0 | 2 | 0 | 0 | 0 | 0 | 1 | 0 | 3 |  |
|  | Total | 28 | 9 | 92 | 18 | 14 | 12 | 12 | 13 | 4 | 202 | 29.8 |
| Americas | Brazil | 26 | 7 | 6 | 0 | 2 | 3 | 9 | 7 | 0 | 60 |  |
|  | Haiti | 1 | 1 | 1 | 0 | 1 | 0 | 0 | 2 | 0 | 6 |  |
|  | Mexico | 0 | 1 | 1 | 0 | 1 | 0 | 0 | 0 | 0 | 3 |  |
|  | Honduras | 1 | 0 | 1 | 0 | 0 | 0 | 0 | 0 | 0 | 2 |  |
|  | Nicaragua | 1 | 0 | 0 | 0 | 0 | 0 | 0 | 1 | 0 | 2 |  |
|  | Chile | 0 | 1 | 0 | 0 | 0 | 0 | 0 | 0 | 0 | 1 |  |
|  | Guatemala | 1 | 0 | 0 | 0 | 0 | 0 | 0 | 0 | 0 | 1 |  |
|  | Total | 30 | 10 | 9 | 0 | 4 | 3 | 9 | 10 | 0 | 75 | 10.9 |
| Middle East | Iran | 8 | 0 | 0 | 1 | 0 | 0 | 1 | 2 | 0 | 12 | 1.8 |
| Cross region |  | 10 | 6 | 16 | 2 | 1 | 3 | 0 | 0 | 6 | 44 | 6.5 |
|  | GRAND TOTAL | 115 | 106 | 236 | 69 | 37 | 28 | 30 | 39 | 18 | 678 |  |
|  | Percent of total | 17.0 | 15.6 | 34.8 | 10.2 | 5.5 | 4.1 | 4.4 | 5.8 | 2.7 | 100.0 |  |
